# Plan of Amelioration of the Medical Profession, &c.

**Published:** 1829-07-01

**Authors:** 


**Plan for the Amelioration of the Medical Profession, &c. By Alexipharmacus.***

In the little anonymous pamphlet which lies before us, there are many just observations on the evils existing in the profession—and several plans of amelioration proposed. We have, on many occasions, adverted to the evils; but now begin to despair of any remedy. The wild REFORMERS have armed their adversaries with impregnable weapons, for some years to come; but still, we should not let these important matters be forgotten. The system of apprenticeship—the licensing of general practitioners by the Apothecaries' Company—and the mode of remuneration, are the three great grievances which call for reformation. And first, in respect to the "HALL."

"In order, then, to remedy, as nearly as possible, the inconveniences which appertain to this department of the Profession, and which have produced such loud and just complaints, it appears both reasonable and proper that the Society of Apothecaries should resign to the Royal College of Physicians, the task of examining candidates for Licences to practise the Art of Medicine, provided that body would be willing to take upon them the care and responsibility thereof: or, that they petition the Legislature to enable them to alter their existing charter, whereby they are constituted a trading body, and give them such other powers as in its wisdom shall seem meet and suitable to the improved condition of society, and as shall take from them altogether the character of a trading or mercantile body.*

"That they, also, continue to exercise the Science of Practical Chemistry, in the Laboratory belonging to their Hall, and to make such compounds and preparations as are permitted, or commanded to be prepared by Apothecaries, in the Pharmacopœia of the Royal College of Physicians. That, instead of making sale and profit, by wholesale or retail, upon the compounds so prepared, these be disposed of at cost price to such Practitioners as have obtained licences, and who may be disposed to take the same off their hands.—They may, also, do much good, by presenting an assortment to such meritorious students as pass through their examinations with credit, and have but small

* Octavo, pp. 17, price 1s. 1829.

* "The Company of Apothecaries are not likely to exert themselves for the general interest of their licentiates, or the public, while they find an exclusive interest in their trade; the which, so long as it is carried on for profit and emolument, ought certainly to separate them from all participation in the practice of Physic, since it tends to identify them with Druggists."

means of commencing their professional career; or otherwise dispose of them to public charities, as they think proper.

"That they should at all times, nevertheless, retain in their possession sufficient for the supply of His Majesty's army and fleet, in case of emergency; with such an assortment of simples, as are necessary to the forming a perfect collection of specimens of the Materia Medica, for the inspection of students, whereby those who have not other opportunities, may at all times gain a knowledge of the form, appearance, and sensible properties, of all substances proper to be used in the *cure* of diseases."

Whatever reason there may be in the first part of the foregoing extract, respecting the transference of the examinations from Apothecaries' Hall to the College of Physicians, we cannot imagine how Alexipharmacus could propose such a wild and unjust scheme as that of robbing a Company of its unquestionable right to "sale and profit" of its goods! Does not the author see what an injustice this would be to other chemical laboratories—for who would go to them, when they could get medicines at the Hall for "cost price."? The author's mind does not appear to be quite made up respecting apprenticeships. He thinks the license should be open (and we agree with him) to all who can stand a proper examination, whether they have been previously apprenticed or not.

"And, provided the means I propose for reducing the quantum of material consumed in the practice of physic to its minimum, shall be brought into operation, there will be less reason to regret the shortening of the term of apprenticeship, or its total abolition; as one reason for so pertinaciously insisting thereon, will then be removed, viz. that which occasions the anxiety of some Apothecaries to secure a good living automaton in their shops, for the period of five or seven years; not but that many good reasons might be adduced for continuing a judicious restraint on the liberties of youth, during the few first years of their initiation into a Profession involving such a high degree of moral responsibility. It is desirable, moreover, in order to discipline the mind to habits of accuracy, business, and study.

"It is the abuse of this otherwise most valuable portion of a student's life, which might be called the seed-time of the mind, that has led to so much just complaint against the principle of apprenticeships in a liberal profession."

After descanting on the evils of remuneration by charges on medicines, the author next proposes his formula for the remedy.

"The most proper principles on which to regulate the remuneration of general Practitioners, appear to be the following:—

"First, for the poorest classes of the community (not within the reach of public Institutions, without risk of an aggravation rather than a relief of their suffering,) and that class whose native modesty and decent pride often occasion them much distress, and who are most particularly worthy of encouragement;—a system of œconomy, somewhat on the same principle as public Institutions, Clubs, or Benefit Societies, supply necessary medical attendance and medicines.

"That which is necessary may be supplied in the simplest form, (on an average calculation,) for one guinea per annum for each person, in sickness or in health, taking the year one time with another, and attendance within certain limits. To every father of such a family, I would give authority to sign a transferable order, to be in force for a year, on receiving in advance the above sum; and to individuals whose necessities do not permit them to do this, attendance should be granted, on receipt of one shilling per week, or an assurance in writing from a responsible friend or neighbour, for the due payment; but though I think it right to mention this, it is not necessary to insist on it, as any person having paid for an order, could transfer the same to such poor neighbours,—the Practitioner to inform the lender, as soon as the patient no longer needs it. To prevent all unnecessary delay and inconvenience, which would result in seeking and obtaining the order, a *first* application always to be attended to *without* it; at the second visit to bring one; or if any difficulty remains, the Practitioner to supply the name of some person on whom the patient may call.

"And thus I would recommend to all Medical Practitioners to consider the condition of their poorest neighbours, and keep records of all that happens to them. These means, pursued systematically, would present no difficulty to any well-informed Student, at the commencement of his practice.

"The additional time and labour thus bestowed, to be compensated for, as shall hereafter be pointed out. The General Practitioner, in this more extended application of the term, would have no pretext for neglecting his poorer, for his richer neighbours. It will be remarked, that no *unnecessary* time, labour, or expense is required of him. He should be the Physician of the poor as well as the rich. There is no need of empirical displays of presumed superior talent, or for wiredrawn reasonings, in order for a medical man to do his straight-forward duty to every sick person who applies to him.

"All this may be effected, without in any degree wounding the feelings of the really deserving and necessitous, almost without their knowledge, as all that can be required of them, is to attend to the appointments made with them, and the prescription and advice of their Medical friend.

"To persons a degree superior, whose condition, though not affluent, is such as to set them above an application for gratuitous Medical attendance; and who may be supposed to possess a laudable pride of independence, in paying for all things to the utmost of their means;—the mode that occurs to me, as likely to be agreeable to all parties, is by graduated fees for each necessary visit or consultation, from 2*s*. 6*d*. upwards, as far as 10*s*. according to the circumstances, and more in no case to be demanded; necessary medicines to be supplied without any additional charge, except in cases of very expensive medicines prescribed by a Physician, or with the consent of the patient, when found to exceed the amount of the fee agreed on: the minimum being fixed at 2*s*. 6*d*. prompt payment in all cases.

"This scheme, if examined in all its bearings, in connection with other projects for the amelioration of the condition of the poor, will be found to bring home to every man's door, in the true spirit of benevolence, that which his necessities require."

Although we believe that some measure analogous to the above must ultimately be adopted by the general practitioners, yet Alexipharmacus does not appear to know the difficulties, nearly insurmountable, which must attend the measure. While the office of prescribing and that of dispensing continue united in the same person, discontent will be experienced from the multitude. If much medicine be sent, and no charge made for attendance, then the patient will grumble at the quantity which he is obliged to swallow:—if, on the other hand, little medicine be ordered, and visits charged, the observation will be, "Oh, the medicines are economised, and the visits are spun out, for obvious reasons!" Nothing but a complete separation of prescription from pharmacy, can free the practitioner from irksome office and ungenerous suspicion. But then what a sacrifice must be made! the whole profits of the dispensing branch will be thrown into the hands of the chemists, who will become what the apothecaries were 30 or 40 years ago. Besides, unless pharmacy were actually prohibited by law, (which would be a very severe enactment) a considerable proportion of general practitioners would always be found, who would unite the prescription of medicines with the preparation of them, and thus secure the monopoly of the lower classes of society.

The author of this pamphlet has spoken in the most handsome, not to say eulogistic, manner of physicians.

"The admiration and respect due to Physicians as a class of gentlemen, and scholars, associated for the advancement of the science of Medicine, and for the benefit of the human race, cannot be mentioned in terms sufficiently expressive. In this class is concentrated a galaxy of learning and skill; and from their influence alone is any *great* improvement of the Science to be expected.

"The education expected to be acquired by them is far too expensive for a sufficient number to meet the wants of the community.

"The Physician, by his good fortune, education, and habits of study, becomes acquainted with the learning, theory, and practice of ancient and modern times, relating to diseases; and by his observations on the living and the dead, in his diurnal avocations, informs himself fully of their nature. He also studies extensively the remedial agents found abundantly spread throughout the world by the wise and beneficent Creator, in the animal, vegetable, and mineral kingdoms, and abstracts from them certain qualities, which, by his own experience and that of others, he finds capable of producing all the various effects that can be desired or expected from them, consistent with the immutable laws to which the human frame is subject.

"All this may be effected by a person blessed with the means and opportunity of a life of exemption from inferior pursuits, spent in this way. But this is the lot of comparatively few; far too few for the demands of general society, whose wants are so great and means too much limited, to command the regular attendance of a Practitioner so well educated."

After such a panegyric, which, we confess, is rather more courteous than strictly true, we were not a little surprised to find that Alexipharmacus was not merely an expeller of physic, but of physicians! The portrait which he has drawn above is fit only to hang over mantle-pieces, or in the halls of colleges. It is too ideal for real life. The physician is to leave the drudgery of practice to the general practitioner, and is to be set apart, as a kind of abstract being, denominated a "GUARDIAN of the PUBLIC HEALTH!"

"It is desirable to diminish the sum of human suffering, consequently the occasions for practice; and it is not difficult to contemplate such an improved state of society, as may nearly supersede the active duties of Physicians, and place them merely in the light of *guardians* of the public *health*.

"The Physician thus exhalted, and set apart from his more humble and active colleague, is not, however, the less useful member of society. The term seems to convey an assurance of the possession of every requisite for the fulfilment of the duties of their enviable and honourable station. The activity of their well-stored minds will always stimulate them to works of the highest importance and utility in their profession; but it is to be lamented, that the fruit of their labours cannot be brought within the reach of every one, on account of the expense attending them; besides this singular and unfortunate consequence, that the more useful they are in themselves, the less likely are they to be universally adopted; and even when resorted to, to be appreciated by the persons most benefited; and, moreover, the general Practitioner, having the charge of his own interests, is oftentimes unwilling to adopt expensive and highly useful medicines, as upon medicines only can he remunerate himself; the interest of the Practitioner being opposed to good practice.

"Though it is true, the adoption of the system of fees would not immediately remove this evil; and a person so disposed, would, (if one may imagine a man so very blind to his real interests,) still have power to protract a case; yet the general purification of the profession, which it will be seen, on a review of the whole plan, must result in process of time, will remove every temptation or motive to mal-practice, if such could for a moment be supposed to operate or exist in the mind of any well-educated man."

There are several passages in the above extract which we do not pretend to understand; but we suspect that there are not a great number of physicians in this metropolis, or, indeed, in these isles, who are over-anxious to be "thus exalted and set apart from their more humble and active colleagues." But our limits are exceeded, and those who are curious to know the various speculations and proposals of Alexipharmacus must consult the original.

